# Development of an sEMG sensor composed of two-layered conductive silicone with different carbon concentrations

**DOI:** 10.1038/s41598-019-50112-4

**Published:** 2019-09-30

**Authors:** Shunta Togo, Yuta Murai, Yinlai Jiang, Hiroshi Yokoi

**Affiliations:** 1 Department of Mechanical and Intelligent System Engineering, Graduate School of Informatics and Engineering, The University of Electro-Communications, Tokyo, Japan; 2 Center for Neuroscience and Biomedical Engineering, The University of Electro-Communications, Tokyo, Japan; 3 Beijing Innovation Center for Intelligent Robots and Systems, Beijing, China

**Keywords:** Biomedical engineering, Electrical and electronic engineering

## Abstract

To achieve robust sEMG measurements in an EMG prosthetic system, this study proposes a surface electromyogram (sEMG) sensor with a novel electrode structure composed of two-layered conductive silicone with different carbon concentrations. We hypothesized there is an optimal carbon concentration for achieving a large sEMG amplitude with robustness to external perturbation, and we empirically determined this optimal concentration. We produced fourteen sets of electrodes, with the weight ratio of carbon to silicone ranging from 1.7% to 4.0%. Using these electrodes, the user sEMG and electrical properties of the electrodes were measured. An external perturbation was applied on one side of the electrode to introduce a condition of unbalanced contact to the sEMG sensor. We defined an index of robustness for the sEMG sensor based on the signal-to-noise ratio in the balanced and unbalanced contact conditions. Based on the results of the robustness index, two optimal carbon concentrations, at weight ratios of 2.0%–2.1% and 2.6%–2.7%, were observed. Moreover, the double-peak property was correlated to the capacitance. Our results clearly demonstrate an optimal carbon concentration for robust sEMG measurements, and suggest that the robust measurement of sEMG is supported by the capacitance component of the sensor system.

## Introduction

Electromyography (EMG) is often used as an input signal to intuitively control a powered prosthetic hand^1–3^. EMG is a biological electric potential generated when a muscle is activated. Conventionally, the magnitude^4^ or rate of change^5^ of EMG has been used to control a prosthetic hand. However, the conventional method can only control a few kinds of motion^6,7^; therefore, researchers have developed control methods based on pattern recognition^8,9^ and regression^10,11^. In any method, stable EMG measurements are important for stable control of an EMG prosthetic hand^12–14^. The EMG is generally measured by the following two methods: invasive and non-invasive. In the invasive method, the EMG is directly measured inside or near the muscle by inserting an electrode^15–17^. This method can precisely measure individual muscle activity without crosstalk, but the invasion is too substantial for users and is not suitable for long-term use. Therefore, the invasive method is not suitable for everyday-use devices. In the non-invasive method, the EMG is measured on the skin surface; hence, the surface EMG (sEMG) can be safely measured with a lighter burden for the user. Therefore, the non-invasive method is suitable for EMG prosthetic hand devices. In conventional physiological experiments in a laboratory or medical environment, a “wet electrode” (such as a silver/silver chloride (Ag/AgCl)-type electrode), is generally used to measure a high-precision signal^18,19^. The wet electrode reduces the electrical impedance (EMG signal is considered as an alternating-current signal) between the skin and the electrode to a smaller value than that of a “dry electrode.” For everyday-use devices, the wet electrode is not suitable because of the burden of attachment and running costs. Therefore, many commercially available sEMG prosthetic hand systems adopt a dry electrode. In many dry electrodes, a metallic material is exposed on the surface and makes contact with the skin^19,20^. An sEMG sensor with a metal electrode can be easily used in everyday life. However, long-term use of the metal electrode is difficult because of its rigidity, and some users are allergic to the metal. Therefore, a dry-type sEMG sensor without contact between the metal and the skin is required.

Previously, researchers developed dry electrodes with soft materials^21–24^. We also developed sEMG sensors using electrodes composed of a conductive fabric^25^ and conductive silicone^26^; these sensors were applied to control numerous types of powered prosthetic hands and arms^27–30^. Electrodes composed of conductive fabric has superior flexibility but causes discomfort to the user, especially for children, because of a metallic element in the conductive fabric^31^. The electrode composed of conductive silicone was a hybrid type of electrode, and both conductive silicone and gold-coated metal wire contacted the skin. The hybrid electrode system can robustly measure the sEMG both under dry and wet skin conditions. However, the exposed gold-coated metal wire remains to present a drawback. Moreover, when the sEMG sensor is used in an EMG prosthetic hand system, the sEMG sensor is disturbed by vibrations in the socket and the body motion of the user, which reduces the controllability of EMG prosthetic hands depending on the pattern recognition technique^32–34^. The disturbance to the sEMG sensor also results in a change of contact force between the skin and electrode. This change of contact force results in an unbalanced contact for the electrodes and an unstable impedance balance^35–38^, leading to unstable sEMG measurements. To achieve stable sEMG measurement in the EMG prosthetic hand system, the problem of unbalanced contact in the electrodes must be resolved.

For the electrode of the sEMG sensor to be suitable for an EMG prosthetic hand system, (1) the sEMG must be robustly measured when the contacts of both electrodes are stable, and (2) the measurement ability of the sEMG cannot undergo substantial changes, even for an unbalanced contact in the electrodes. To achieve requirement (1), the contact impedance between the skin and the electrode should be small^39^. To achieve requirement (2), the contact impedance should be larger than the change in contact impedance between the balanced and unbalanced electrode contact conditions. According to the above two requirements, an electrode suitable for sEMG measurement would have an optimal impedance under this trade-off relationship. Conductive silicone is composed of a mixture of silicone and carbon black powder^26^. Hence, changes in carbon concentration leads to changes in the electrical impedance of the conductive silicone. Metallic materials can be used to connect the conductive silicone and the amplifier circuit. However, we found that the contact impedance between the metal and the conductive silicone with low carbon concentrations is much higher than that between the conductive silicones (see Supplementary Information). Therefore, conductive silicone with a high carbon concentration should be connected to the amplifier via a metallic material, and the conductive silicone in the electrode, which has a low carbon concentration, should come in contact with the skin and the conductive silicone with the high carbon concentration. We propose a two-layered electrode consisting of conductive silicone with different carbon concentrations. To develop the proposed electrode, we must find the optimal carbon concentration for the conductive silicone of the electrode under the trade-off relationship.

The aim of this study is to design an sEMG sensor structure with two-layered electrodes and to find the optimal carbon concentration of the conductive silicone in contact with the skin. We produced electrodes with different carbon concentrations and measured the corresponding sEMG. The signal-to-noise (SN) ratio was defined as the ratio of sEMGs measured in relaxing and grasping states; the SN ratio is used to quantitatively evaluate the sEMG measurement ability of each electrode. To generate unbalanced and balanced electrode contacts artificially, we adopted conditions in which one electrode is or is not externally pressed. We evaluated the difference in SN ratio between the non-pressed and pressed conditions. An index of robustness for the sEMG measurement was defined using the value of the SN ratio and the change in SN ratio. We expected the index of robustness to show a single peak at a specific carbon concentration. Moreover, we measured the electrical properties of the electrode with skin contact and evaluated the relationship between the electrical properties and the robustness index.

## Results

**Figure 1:**
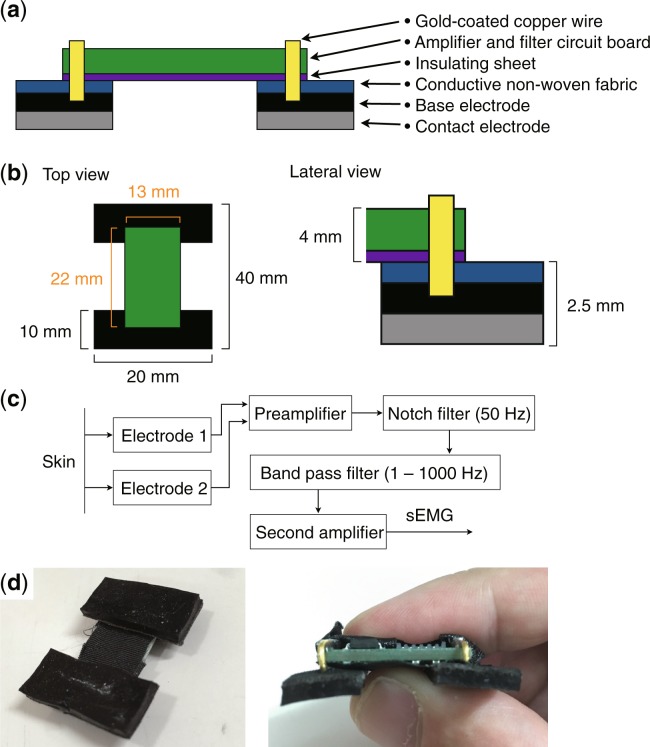
Our developed sEMG sensor with two-layered conductive silicone. (**a**) Design of the sEMG sensor. The two-layered base and contact electrodes are attached to the amplifier and filter circuit board by conductive non-woven fabric and a gold-coated copper wire. An insulating sheet is placed between the circuit board and the electrodes. (**b**) Size of the sensor. The total size of the proposed sEMG sensor is 20 × 32 × 6.5 mm. (**c**) Details of the amplifier and filter circuit. (**d**) Photographs of the developed sEMG sensor.

**Figure 2:**
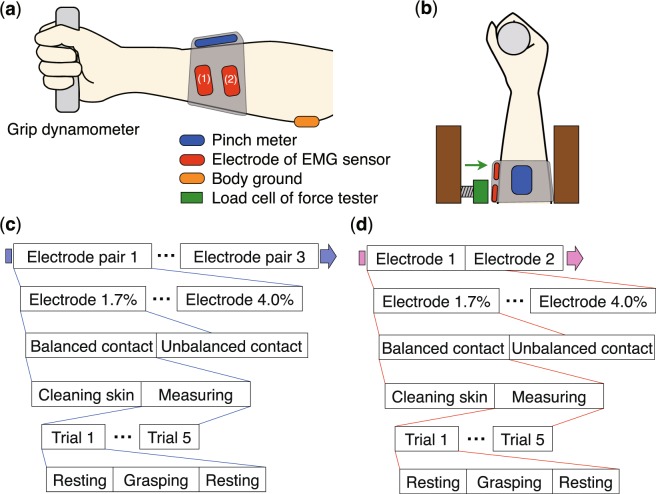
Experimental setup for measuring the sEMG signal and electrical properties of the electrodes. Lateral view (**a**) and top view (**b**) of the apparatuses. The grip dynamometer measures the grip force of the subject. The pinch meter is used to verify contact between the sEMG sensor band and the skin. The pinch meter and sEMG sensor are attached to the arm by a rubber band. The load cell applies an external force to one side of the sEMG sensor electrodes. Under this condition, the sEMG signal (**c**) and electrical properties (**d**) are measured.

We developed an sEMG sensor with two-layered electrodes, as shown in Fig. 1 (see Methods). The two-layered electrodes consisted of a contact electrode (contacting the skin) and a base electrode (contacting the gold-coated copper wire and the contact electrode). We empirically investigated the optimal carbon concentration of the contact electrode. Further, we measured the sEMG and electrical properties when the subject was relaxed and when the subject grasped a grip dynamometer, as shown in Fig. 2a. The unbalanced contact condition was introduced by externally pressing one side of the electrode (Fig. 2b). The SN ratio was defined as the ratio of the sEMG measured under the grasping and relaxing conditions.

**Figure 3:**
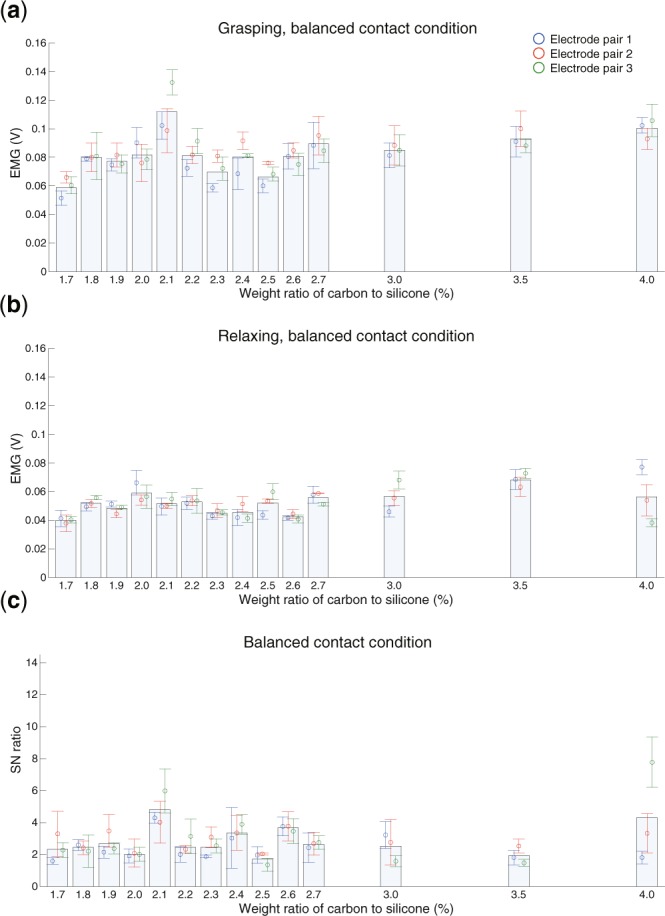
Performance of sEMG measurement under the balanced contact condition. The horizontal axis presents the weight ratio of carbon to silicone. The vertical axis indicates the sEMG amplitude when the subject grasps the grip dynamometer (**a**) and relaxes (**b**) and the SN ratio (**c**). The bars and circles indicate the average across all the electrodes and average data for each electrode pair, respectively.

Figure 3 shows the results of the sEMG measurement and SN ratio for the balanced contact condition. The sEMG for the grasping motion in the balanced contact condition was the largest for the electrode with a carbon concentration of 2.1% (Fig. 3a). In addition, the variance across the electrode pairs was large for the electrode with the maximum carbon concentration of 4.0%. Figure S3 shows typical raw data of the measured sEMG waveform. We chose 2.1% and 2.5% electrode pairs as they showed a large difference in sEMG amplitude in Fig. 3a. This result clearly demonstrates that the carbon concentration in the electrode largely affected the performance of sEMG measurement and that a high carbon concentration did not necessarily result in a better measurement performance.

**Figure 4:**
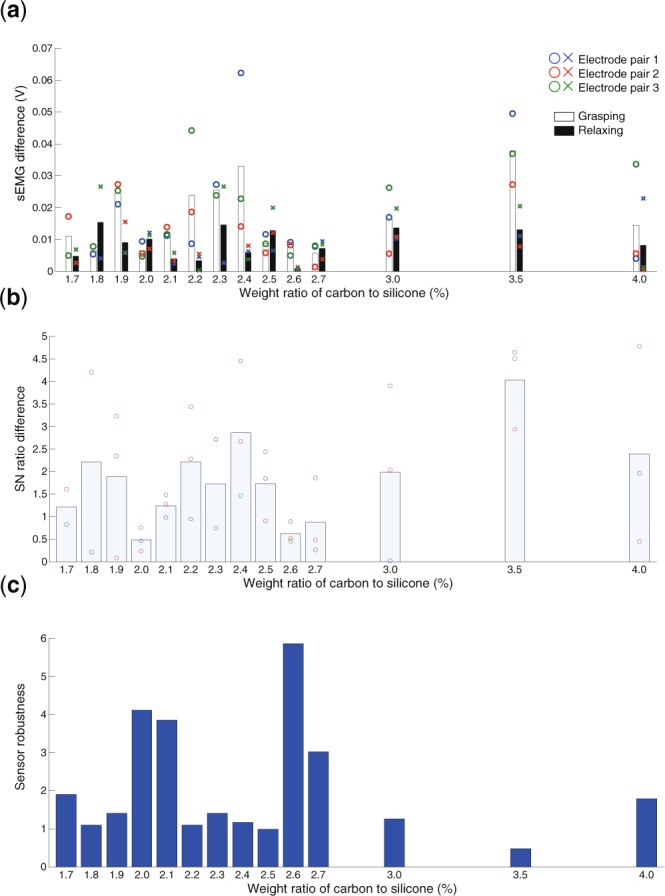
Comprehensive evaluation of sEMG measurement performance. The horizontal axis displays the weight ratio of carbon to silicone. The vertical axis indicates the sEMG difference (**a**) and SN ratio difference (**b**) for the balanced and unbalanced contact conditions and the index of robustness for the sEMG measurement (**c**).

For the SN ratio, a peak was observed near concentrations of 2.1% and 2.6% (Fig. 3c). Moreover, a large SN ratio was observed at 4.0%, but the variance across the electrode pair was also large. The above results clearly demonstrate that the carbon concentration of the contact electrode influences the sEMG measurement under the balanced contact condition. Figure S4 shows the results of the sEMG measurement and SN ratio for the unbalanced contact condition. Based on the results for the balanced contact condition, it is clear that pressing one side affected both the measured sEMG and SN ratio. This result shows that the unbalanced electrode contact leads to unstable sEMG measurements. Figure 4a shows the absolute value of the difference in measured sEMG between the balanced and unbalanced contact conditions, and Fig. 4b shows the corresponding SN ratio results. The variation in the measured sEMG for the grasping motion was large, particularly at carbon concentrations of 1.9%, 2.2%–2.4%, and 3.5% (Fig. 4a). The variation in the SN ratio showed similar trends. Moreover, the variation in the SN ratio was small at carbon concentrations of 2.0% and 2.6%, meaning the results indicated two optimal carbon concentrations that are stable against external perturbation (Fig. 4b). We defined the robustness index of the sEMG measurement as the ratio of the SN ratios for the balanced contact condition and its change between the two conditions (see Eq. 3 in Methods). Figure 4c shows the index of robustness *I*_*r*_. The results clearly show two peaks at 2.0%–2.1% and 2.6%–2.7%. The above results demonstrate that the contact electrodes with carbon concentrations of 2.0%–2.1% and 2.6%–2.7% can robustly measure sEMG.

**Figure 5:**
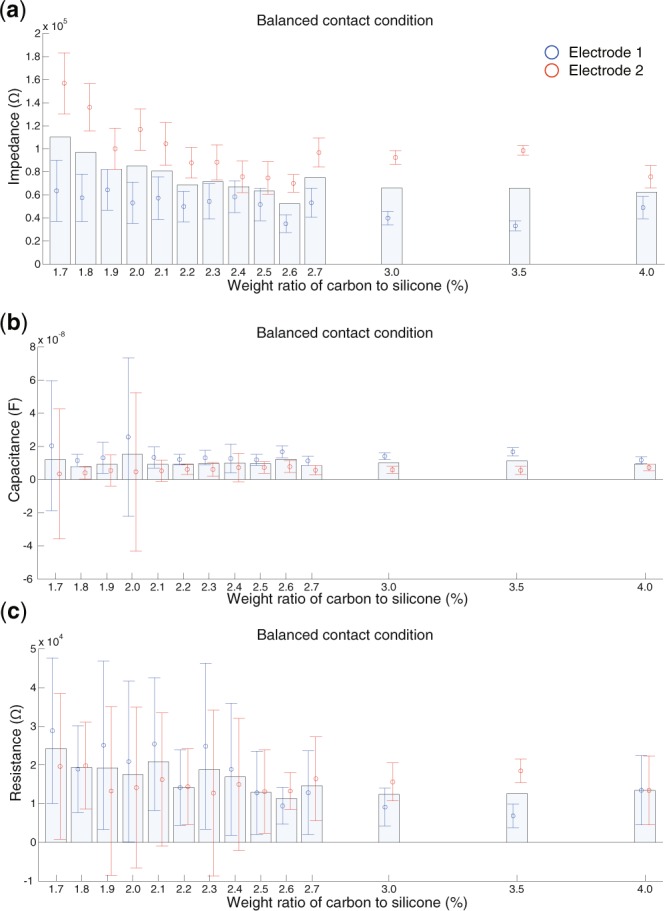
Electrical properties of electrodes under the balanced contact condition. The horizontal axis presents the weight ratio of carbon to silicone. The vertical axis indicates the impedance (**a**), capacitance component (**b**), and resistance component (**c**). The bars and circles indicate the average across all the electrodes and average data for each electrode, respectively.

Figure 5 presents the electrical properties measured under the balanced contact condition. The impedance shows a decreasing trend as the carbon concentration increases, with a minimum value at approximately 2.6% (Fig. 5a). The capacitance component (Fig. 5b) and the resistance component (Fig. 5c) both show relatively larger variance values than the impedance (Fig. 5a). The capacitance component does not show a clear trend, but one electrode (blue circle) shows a relatively high value at 2.0% while the other shows a similar value at 2.6%. The resistance component shows a minimum value at 2.6%, as observed for the impedance (Fig. 5c). The above results demonstrate that the carbon concentration affects the electrical properties. Moreover, the characteristic points were observed around the carbon concentrations showing the two peaks of the robustness of sEMG measurement.

**Figure 6:**
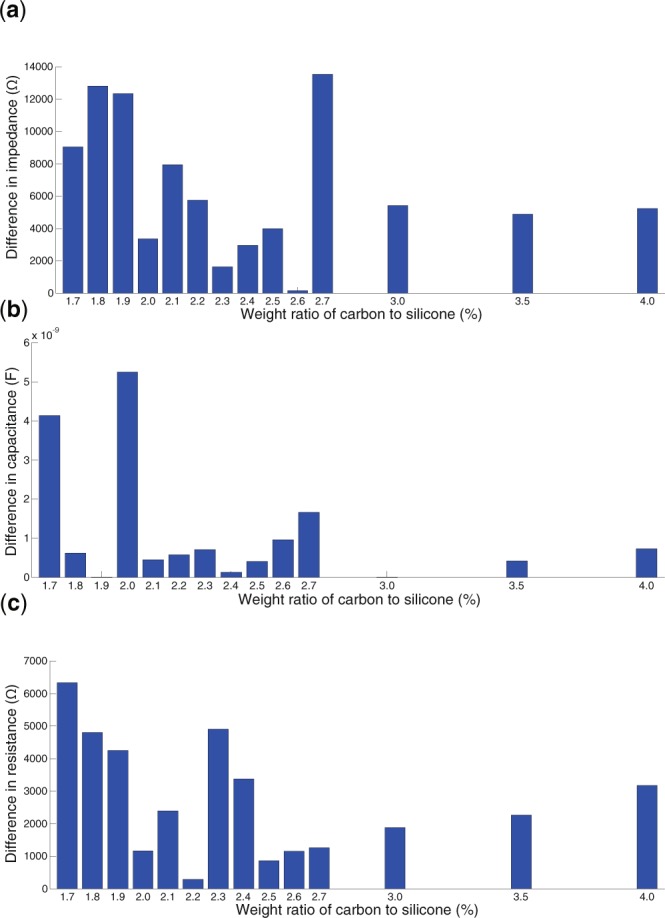
Comprehensive evaluation of the electrical properties of the electrodes. The horizontal axis displays the weight ratio of carbon to silicone. The vertical axis indicates the difference in impedance (**a**), capacitance component (**b**), and resistance component (**c**) between the balanced and unbalanced contact conditions.

Figures S5 and 6 show the electrical properties for the unbalanced contact condition and the absolute difference in electrical properties between the balanced and unbalanced contact conditions, respectively. The external perturbation did not affect the dependency of the electrical properties on the carbon concentration. In the sEMG measurements, two peaks were observed around concentrations of 2.0%–2.6%. In the electrical property measurements, differences in the impedance (Fig. 6a) and resistance (Fig. 6c) between the balanced and the unbalanced contact conditions around 2.0%–2.6% were relatively small. Conversely, differences in the capacitance around the same range were relatively large (Fig. 6b). The above results demonstrate that the electrical properties would influence the index of the sensor’s robustness, depending on the carbon concentration. Next, we verify the relationship between the electrical properties and the robustness of sEMG measurement.

**Figure 7:**
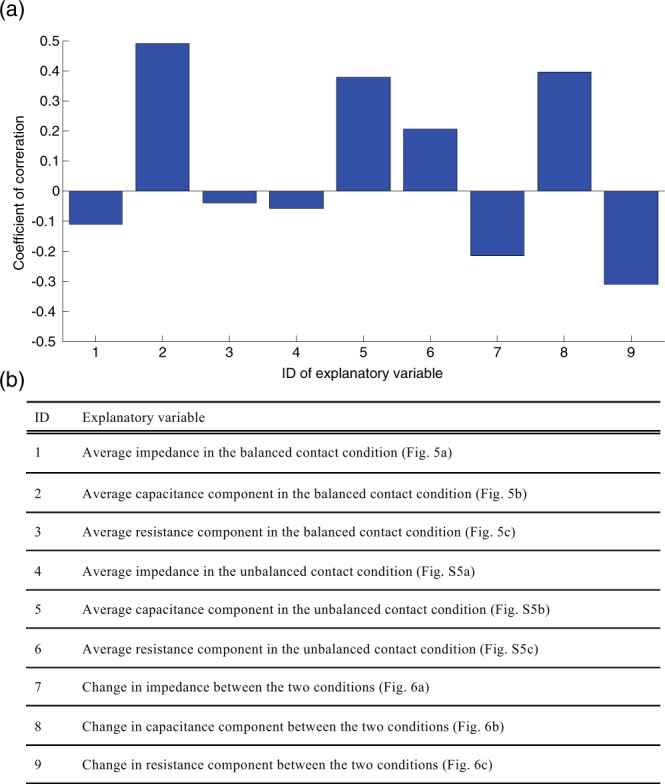
Correlation analysis of the index of robustness for sEMG measurements and the electrical properties. (**a**) Correlations between the index of robustness for sEMG measurements (Fig. 4c) and the explanatory variables. The horizontal and vertical axes display the ID of the explanatory variables (**b**) and the coefficient of correlation, respectively.

Finally, analysis results regarding the correlation between the change in robustness of the sEMG measurement (Fig. 4c) and change in electrical properties (Figs 5, 6 and S5) are shown in Fig. 7. The highest correlation coefficient was observed for the explanatory variable of capacitance under the balanced contact condition (ID: 2, *r* = 0.49), and the second highest value was observed for the change in capacitance between the balanced and unbalanced contact conditions (ID: 8, *r* = 0.40). The third highest value was found for the capacitance under the unbalanced contact condition (ID: 5, *r* = 0.38). The positive correlation between the sEMG measurement robustness and the capacitance for the balanced contact condition indicates that a greater capacitance corresponds to a more robust sEMG measurement. Remarkably, all three high correlation coefficients were observed for explanatory variables related to the capacitance. The results suggest that the capacitance is the cause of the double-peak nature of the sEMG measurement robustness.

## Discussion

In this study, we assumed there is an optimal carbon concentration for conductive silicone in contact with skin based on the two requirements of sEMG sensors suitable for EMG prosthetic hands, and we experimentally determined the optimal concentration. We produced fourteen sets of electrodes with carbon concentrations ranging from 1.7% to 4.0% and measured the sEMG and electrical properties for each concentration. Moreover, we applied an external perturbation of 20 N to one side of the electrodes for an unbalanced contact with the electrodes of the sensor. The results demonstrate the existence of carbon concentrations that can robustly measure large sEMG amplitudes under the balanced contact condition of the electrodes (Fig. 3c), and that can maintain a stable SN ratio between balanced and unbalanced contact of the electrodes (Fig. 4b). The robustness index for sEMG measurement showed two peaks at carbon concentrations of 2.0%–2.1% and 2.6%–2.7% (Fig. 4c). These results indicate that the conventional approach for sEMG sensors, which reduces the electrode impedance with a higher carbon concentration, cannot obtain robust sEMG measurements. We concluded that the optimal weight ratios of carbon to silicone in this study are 2.0%–2.1% and 2.6%–2.7%.

Initially, we expected that a single carbon concentration peak would be observed based on the trade-off of two requirements for robust sEMG measurement. Contrary to our expectation, the results of the robustness index of sEMG measurement showed two peaks. This double-peak property (Fig. 4c) and the capacitance component in the balanced contact condition showed the highest coefficient of correlation (Fig. 7). Moreover, the three highest coefficients of correlation were related to the capacitance components. These results suggest that the double-peak property is dependent on the capacitance component of the electrode. When the carbon concentration was increased, the capacitance of the electrode increased because the distance between the carbon particles decreased and the surface of the carbon group was large. A high carbon concentration results in a connection of carbon chains in the electrode, leading to a small capacitance. These trends lead to the first peak of robust sEMG measurement. Meanwhile, the contact area of the carbon component in contact with the skin is large for high carbon concentrations, which leads to a high capacitance. The capacitance then saturates beyond a certain carbon concentration. We infer that this mechanism leads to the second peak. The difference in the two capacitance peaks in the electrode and between the electrode and the skin is an important factor. A higher capacitance corresponds to an easier transmission of the alternating current signal, i.e., the sEMG signal. Therefore, it is suggested that the electrical property of the capacitance affects the robustness of the sEMG measurement. However, as the electrical properties of only two electrodes were obtained, it would be difficult to discuss the cause of the double peak further. It is required to test the above inference in future work.

The proposed structure of the electrode is based on the assumption that the contact impedance is small between the conductive silicone components and relatively large between the conductive silicone and the metal. The achievement of a robust sEMG measurement at a specific carbon concentration implies the feasibility of our assumption. Remarkably, under the balanced contact condition, the electrode with a carbon concentration of 2.1% measured sEMGs at an amplitude equal to or larger than that at 4.0%, the highest carbon concentration (Fig. 3a). This result demonstrates that a low electrode impedance is not necessarily optimal and highlights the importance of considering the contact impedance. However, the mechanism governing the contact impedance of the conductive silicone is not clear. In future work, we plan to evaluate the mechanism of the contact impedance of conductive silicone at the molecular level.

## Methods

### Development of the sEMG sensor electrode

The electrode contacts the skin of the user to measure the sEMG. Hence, the electrode should possess non-allergic properties and flexibility for long-term use. In this study, the conductive silicone consisted of a mixture of silicone (TSG-E30, Tanac Co. Ltd., Japan) and carbon black (EC600JD, Lion Specialty Chemicals Co. Ltd., Japan) was used for the electrode^26^.

Based on the *supplementary discussion*, we have proposed a two-layered electrode structure and a sEMG sensor system, as shown in Fig. 1a. The base electrode is placed on the contact electrode (contacting the skin), and the conductive non-woven fabric is placed on the base electrode to avoid rupture of the silicone. The gold-coated copper wire is in contact with the conductive non-woven fabric and the base electrode and connects the electrode and the amplifier and filter circuit board. An insulating sheet is placed between the circuit board and the electrodes. The measured sEMG signal passes through the path of the contact electrode, base electrode, conductive non-woven fabric, gold-coated copper wire, and amplifier and filter circuit. The size of the circuit board was 13 × 22 × 4 mm, as shown in Fig. 1b. The size of the electrode is arbitrary, and was set to 10 × 20 × 2.5 mm for the experiments. The size of the sEMG sensor was 40 × 20 × 6.5 mm. The amplifier and filter circuit is shown in Fig. 1c. The measured sEMG is amplified by a preamplifier using an instrument amplifier AD620 (Analog Devices, USA) with a gain of 40 dB; the signal is then filtered by a notch filter of 50 Hz, and a band-pass filter with a bandwidth of 1 to 1000 Hz, and is again amplified by a second amplifier. The output voltage ranges from 0 to 5 V. Photographs of the developed sEMG sensor are shown in Fig. 1d.

### Experiments to determine the optimal weight ratio of carbon to silicon

We conducted experiments to determine the optimal carbon concentration of the contact electrode for sEMG measurement.

#### Subject

One healthy male subject in his twenties participated in the experiments. The subject did not have any rashes on his skin. All experiments were approved by the ethics committee of the University of Electro-Communications. The participant received explanations of the experimental procedure and gave written informed consent. In addition, all procedures were performed in accordance with the Declaration of Helsinki and the guidelines of the Japan Neuroscience Society and the Japan Society of Clinical Neurophysiology.

#### Apparatus

We measured the sEMG signal and the electrical properties of the electrode using the apparatus shown in Fig. 2. A grip dynamometer (G100, Biometrics Ltd, United Kingdom) was employed to measure the grasping force of the subject. The electrodes of the sEMG sensor were placed on the skin on the flexor digitorum superficialis for the grasping action. A wet electrode (Biorode, Nippon Becton Dickinson Company, Ltd., Japan) was placed on the skin on the medial epicondyle as a body ground. To verify the contact pressure between the skin and the electrode, a pinch meter (P100, Biometrics Ltd, United Kingdom) was used. The electrode and pinch meter were fixed to the arm by a rubber band. The amplifier and filter circuit board was placed on the outside of the rubber band in the experimental condition. An external perturbation was applied to one side of the electrode by pressing the load cell of a force tester (MCT-2150, A&D Co., Ltd., Japan) against electrode (2) from outside the rubber band. We used a data logger (AI0-160802AY-USB, CONTEC Co., Ltd., Japan) to measure the sEMG with a 1000-Hz sampling frequency. We also used an LCR meter (3532-50, HIOKI Co., Ltd., Japan) to measure the electrical properties of the electrode. The probes of the LCR meter were connected to the electrode and the body ground. We measured the electrical properties of the system, including the electrode, skin, and arm, when the EMG was generated.

The measured variables included the impedance, resistance component, and capacitance component; the impedance was represented by an equivalent circuit of a serial connection of the resistance and capacitance components. In the electrical property measurements, the input voltage was 0.1 V, the input frequency was 300 Hz, and the sampling rate was 4 Hz. We produced fourteen sets of contact electrodes with different carbon concentrations; the weight ratios of carbon to silicone were 1.7%, 1.8%, 1.9%, 2.0%, 2.1%, 2.2%, 2.3%, 2.4%, 2.5%, 2.6%, 2.7%, 3.0%, 3.5%, and 4.0%. We produced four electrodes for each carbon concentration (electrodes a, b, c, and d) and three electrode pairs for sEMG measurement (electrode pairs a-b, a-c, and a-d). The weight ratio of carbon to silicone for the body electrode was 4.0%.

#### Procedure

The same subject participated in (1) the sEMG measurement experiment and (2) the electrical property measurement experiment on different days.

(1) sEMG measurement: We measured the sEMG of the subject in a relaxed condition and while grasping the grip dynamometer. The task schedule is shown in Fig. 2c. The fourteen sets of electrodes were sequentially used from 1.7% to 4.0% among the three electrode pairs. We adopted the balanced contact (electrodes were not externally pressed) and unbalanced contact (one side of the electrodes was externally pressed) conditions for each type of electrode. Before the sEMG measurement, the skin was cleaned by alcohol. The sEMG measurement consisted of 5 trials. In each trial, the subject relaxed for 1 s, grasped for 3 s, and relaxed for 2 s. The contact force of the electrode was controlled such that the pinch meter read 5 N. The subject performed a grasping motion such that the grip dynamometer read approximately 10 kgf.

In the unbalanced contact condition, the force tester was controlled at 20 N.

(2) Electrical property measurement: We measured the electrical properties of the electrode as the subject performed a grasping motion while in a stationary condition for 3 s. The task schedule is shown in Fig. 2d. We also adopted the balanced and unbalanced contact conditions for each type of electrode, as in experiment (1). The electrode position, electrode fixation method, grasping force, and external perturbation were the same as in experiment (1).

#### Data analysis

Using the measured sEMG data *E*, we calculated the root mean square (RMS), i.e., rectified sEMG data $\hat{E}$, as follows:

1 $${\hat{E}}_{t}=\sqrt{{E}_{t}^{2}},$$

where *t* denotes the time step. We defined ${\hat{E}}_{g}$ and ${\hat{E}}_{r}$ as time-averaged values of $\hat{E}$ during the grasping and relaxing motion in one trial, respectively. Outlier data were defined as data beyond the range of the mean value ± 2 standard deviations across five trials. We removed the outlier data from the following analysis.

In this study, we defined the SN ratio as the ratio of the sEMG signal for the grasping motion to that for the relaxing motion, calculated as follows:

2 $$\eta ={({\hat{E}}_{g}/{\hat{E}}_{r})}^{2}.$$

We defined ${\eta }_{b}$ and ${\eta }_{u}$ as SN ratios for the balanced and unbalanced contact conditions, respectively. In this study, we defined the robustness index for evaluating a stable sEMG measurement as follows:

3 $${I}_{r}=\frac{{\eta }_{b}}{\sqrt{{({\eta }_{b}-{\eta }_{u})}^{2}}}.$$

When the SN ratio in the balanced condition is high and the difference between the SN ratios for the balanced and unbalanced contact conditions is small, the index of robustness is high.

For the measured electrical properties, we calculated the average value for each trial. To assess which electrical properties affect the sEMG measurement robustness, we analyzed the correlation between changes in the robustness index and the measured electrical properties depending on the carbon concentration. We considered the electrical properties shown in Fig. 7b as explanatory variables.

## Supplementary information

Supplementary information
