# Supplementary material for: Development of an sEMG sensor composed of two-layered conductive silicone with different carbon concentrations
Source: Sci Rep. 2019 Sep 30;9:13996. doi: 10.1038/s41598-019-50112-4 (PMC6768884; doi:10.1038/s41598-019-50112-4)
Supplement: Supplementary file 1 — Supplementary information [file 41598_2019_50112_MOESM1_ESM.pdf]

Development of an sEMG sensor composed of two-layered conductive silicone with different carbon concentrations

Shunta Togo[1,2*], Yuta Murai[1], Yinlai Jiang[3,2] and Hiroshi Yokoi[3,1,2]

[1]Department of Mechanical and Intelligent System Engineering, Graduate School of Informatics and Engineering, The University of Electro-Communications, Tokyo, Japan.

[2]Center for Neuroscience and Biomedical Engineering, The University of Electro-Communications, Tokyo, Japan.

[3]Beijing Innovation Center for Intelligent Robots and Systems, Beijing, China.

*Corresponding author

E-mail: s.togo@uec.ac.jp (ST)

Supplementary information

Supplementary methods

We measured the impedance of the electrode made from silicone (TSG-E30, Tanac Co. Ltd., Japan) mixed with carbon black (EC600JD, Lion Specialty Chemicals Co. Ltd., Japan) as shown in Fig. S1. In the measurement experiments, we used an LCR

meter (3532-50, HIOKI Co., Ltd., Japan) to measure the impedance of the electrode. In all the experiments, the electrodes were placed between the SUS430 boards, and a 100 g load was imposed on the boards. The probes of the LCR meter were connected to the load and the boards. The input voltage, the input frequency, and the sampling rate of the LCR meter were 5 V, 2000 Hz, and 1/30 Hz, respectively.

We carried out three types of measurement experiments. First, we measured the impedance of the electrode made from the conductive silicone with 2% carbon and the conductive non-woven fabric. We repeated the same on the electrode with 4% carbon. The layout for these measurements is shown in Fig. S1a. Finally, we measured the impedance of the “sandwich electrode” in which the conductive silicone with 2% carbon was placed between two electrodes t with 4% carbon (see Fig. S1b). We made four electrodes for each carbon concentration and measured four times for each experimental condition. In the “sandwich” condition, the measured electrode with 4% carbon was fixed, while that with 2% carbon was changed. We used steady state data (30 seconds after the onset of measurement).

Supplementary results

Figure S2 shows the mean impedance data for each experimental condition. The bars indicate the impedance for the experimental conditions shown in Figs. S1a and S1b. This result clearly demonstrates that the impedance of the electrode with 2% carbon was the highest. This substantially dropped by approximately 50% when the conductive silicone with 2% carbon was sandwiched between the silicones with 4%

carbon. The impedance of the electrode with 4% carbon was the lowest. The results of one-way ANOVA (*p*-value: $p = 9.8\times10^{-6} < 0.01$, *F*-value: $F_{(2, 9)} = 53.88$) and *post-hoc* test with the Tukey-Kramer method statistically supported these results.

Supplementary discussion

Figure S2 clearly demonstrated that the impedance of the conductive silicone with 2% carbon, sandwiched between those with 4% carbon, was significantly lower than that of the silicone electrode that has no contact with any other conductive silicones. The difference between the above two cases can be attributed to the contact impedance between the conductive silicone with 2% carbon and the metal. Therefore, the results imply that (1) the contact impedance between the conductive silicone and the metal was relatively large when the carbon concentration was low, and (2) the contact impedance between the conductive silicones was relatively low.

As mentioned in the Introduction section of this paper, the impedance of the electrode must be larger than the contact impedance between the skin and the electrode, but small enough to measure large sEMG amplitudes. Moreover, the electrode must be connected to the amplifier circuit board, but cannot be directly soldered to the board. Hence, we considered using a metal wire (gold-coated copper wire) to connect the electrode and the amplifier. According to the implication (1) stated above, the contact impedance between the electrode contacting the skin (termed the "contact electrode") and the metal was high, which would lead to a reduction of the sEMG signal. However, according to implication (2) stated above and the results shown in Fig. S2, the

impedance of the contact electrode via the conductive silicone with high concentration was low. We termed the electrode with high carbon concentration connecting the contact electrode and the amplifier circuit board as the “base electrode.” To meet the requirements, the contact electrode with a relatively low carbon concentration should be used via the base electrode with maximum carbon concentration. Therefore, a two-layered structure consisting of the contact electrode and the base electrode would be suitable for a stable sEMG measurement.

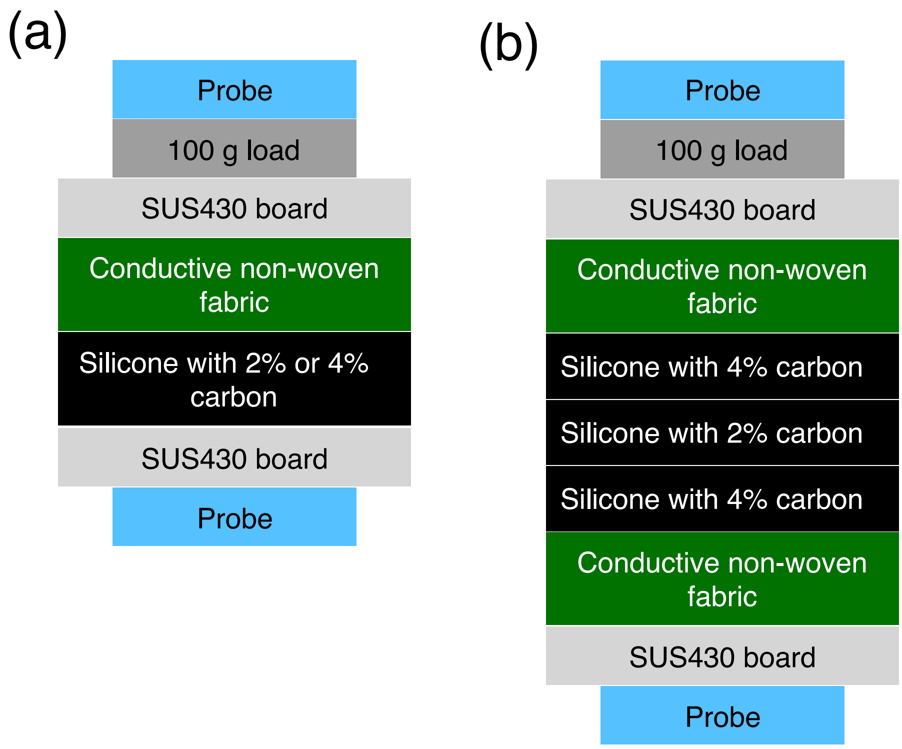

Figure S1 | Measurements of contact impedance of the electrode. (**a**) Measurement condition of the electrode with 2% or 4% carbon concentration. The electrode is placed between the SUS430 boards, with a 100 g load is imposed on the board. The probes of the LCR meter are attached to the metal part. (**b**) The electrode with 2% carbon is placed between the electrodes with 4% carbon.

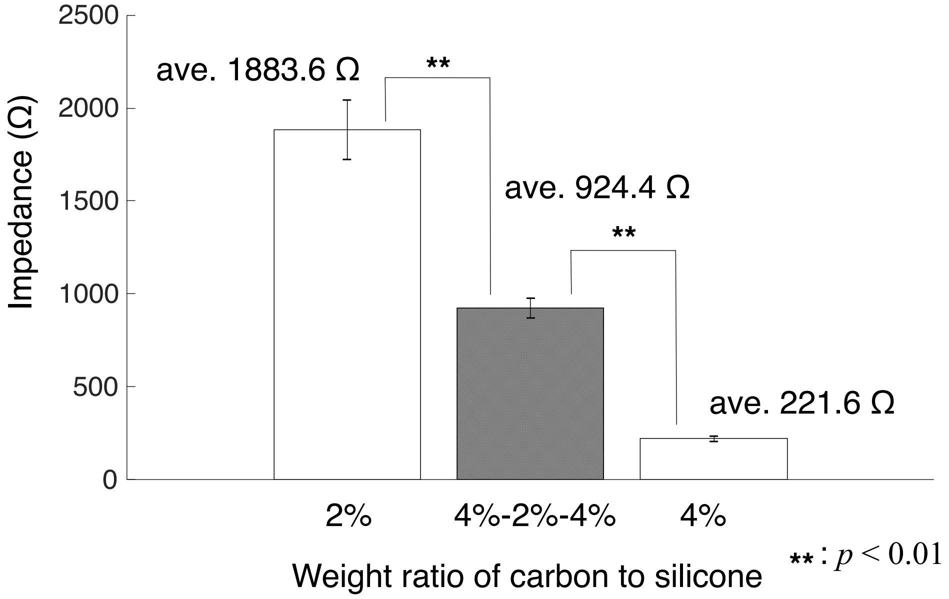

Figure S2 | Impedance of the electrode with different measurement conditions. The horizontal axis denotes weight ratio of carbon to silicone and the vertical axis denotes the impedance. The asterisk indicates significant difference.

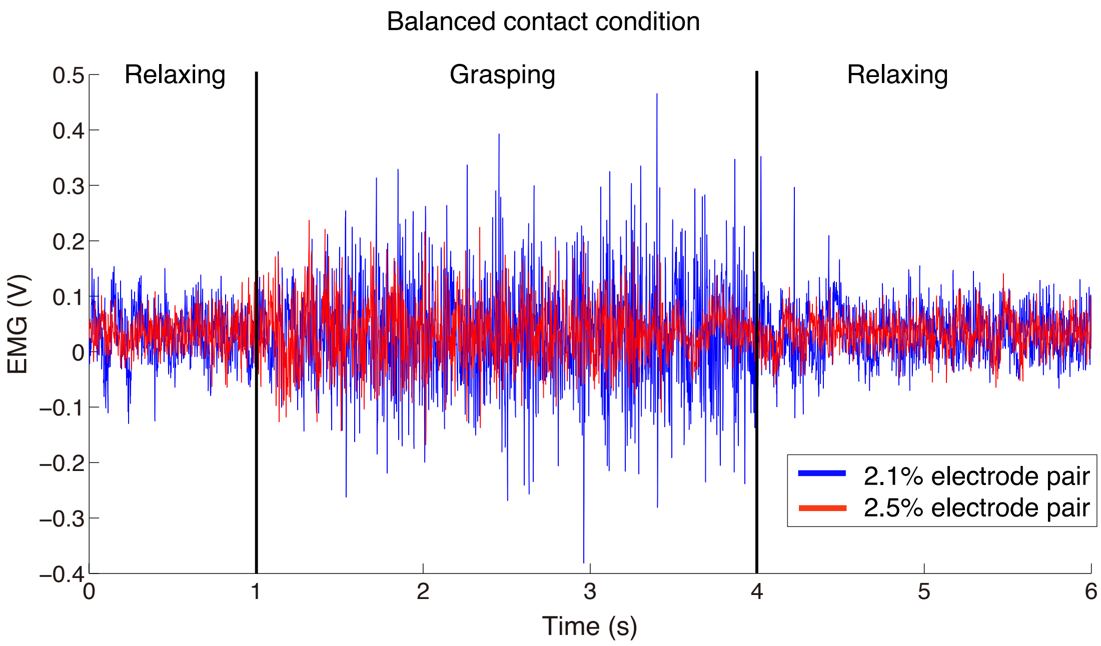

Figure S3 | Typical raw data of the measured sEMG waveform in the balanced contact condition. The horizontal and vertical axes denote time and EMG values, respectively. The blue and red lines indicate sEMG data obtained from the 2.1% and 2.5% electrode pairs, respectively.

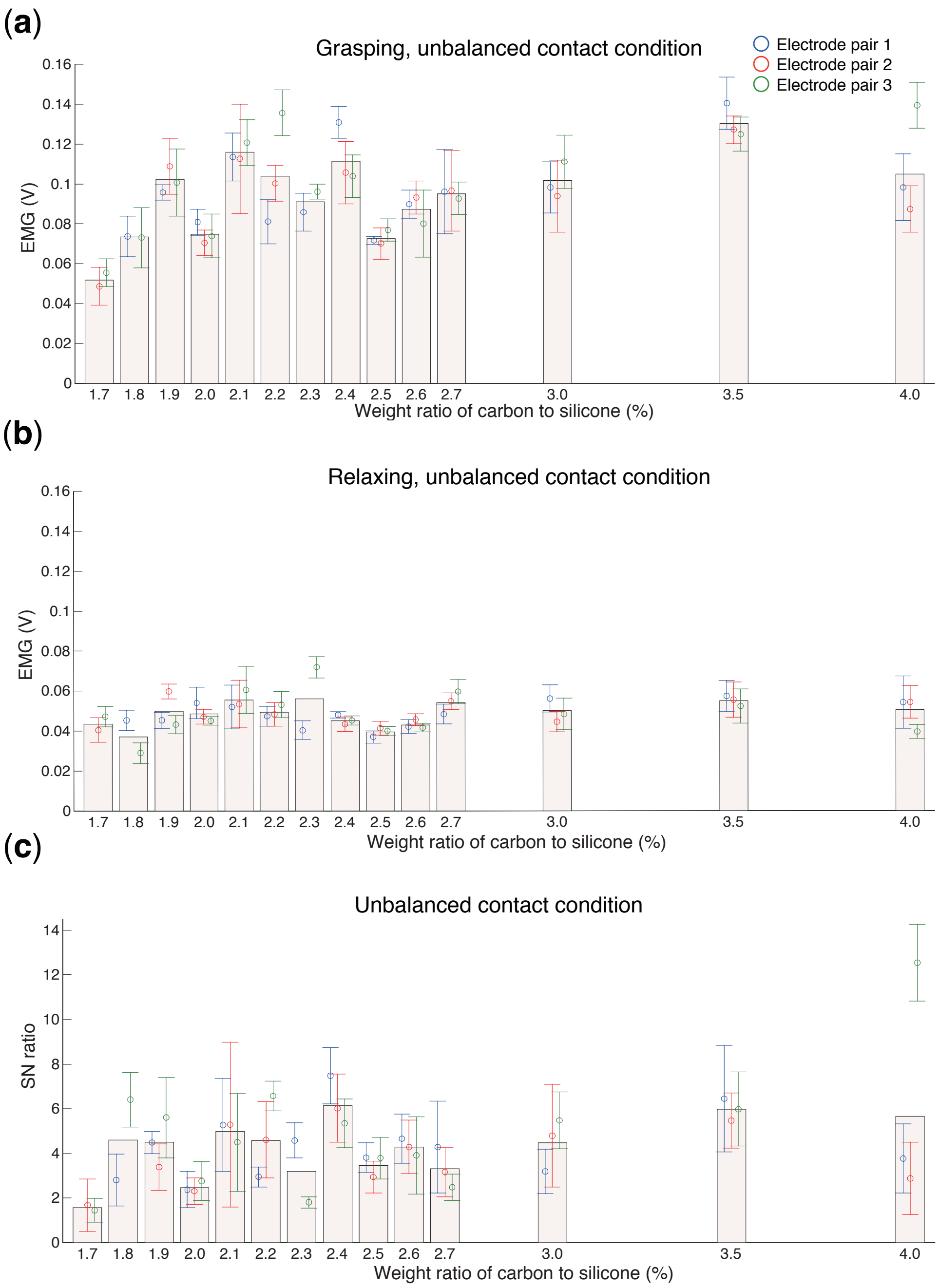

Figure S4 | Performance of sEMG measurement in unbalanced contact condition. The horizontal axis presents the weight ratio of carbon to silicone. The vertical axis indicates the sEMG amplitude when the subject grasps the grip dynamometer (**a**) and relaxes (**b**) and the SN ratio (**c**). The bars and circles indicate the average across all the electrodes and average data for each electrode pair, respectively.

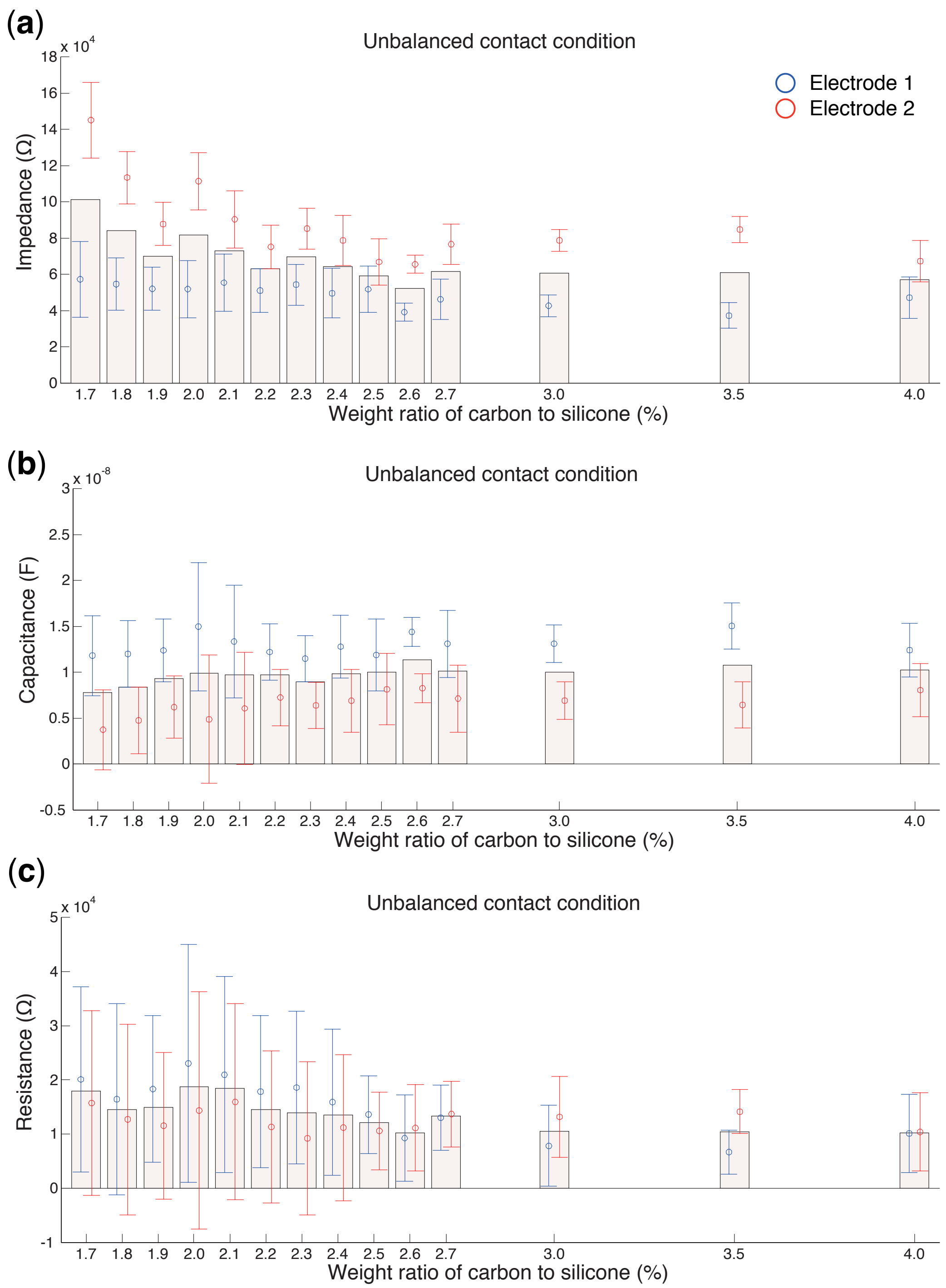

Figure S5 | Electrical properties of the electrode under the unbalanced contact condition. The horizontal axis presents the weight ratio of carbon to silicone. The vertical axis indicates the impedance (**a**), capacitance component (**b**) and resistance

component (**c**). The bars and circles indicate the average across all the electrodes and average data for each electrode, respectively.
